# Prevalence and Molecular Characterization of *Cryptosporidium* Species in Diarrheic Children in Cameroon

**DOI:** 10.3390/pathogens14030287

**Published:** 2025-03-14

**Authors:** Bertrand Sone, Lum Abienwi Ambe, Mireille Nguele Ampama, Constance Ajohkoh, Desmond Che, Julien Alban Nguinkal, Anja Taubert, Carlos Hermosilla, Faustin Kamena

**Affiliations:** 1 Laboratory for Molecular Parasitology, Department of Microbiology and Parasitology, University of Buea, Buea P.O. Box 63, Cameroon; mysonpalle1@gmail.com (B.S.); lum.abienwi@ubuea.cm (L.A.A.); constanceanjohkoh20@yahoo.com (C.A.); cdesmondshu@gmail.com (D.C.); 2 Centre for Research on Health and Priority Pathologies, Institute of Medical Research and Medicinal Plants Studies (IMPM), Yaoundé, P.O. Box 13033, Cameroon; 3 Institute of Parasitology, Biomedical Research Center Seltersberg (B.F.S.), Justus Liebig University Giessen, 35392 Giessen, Germany; anja.taubert@vetmed.uni-giessen.de (A.T.); carlos.r.hermosilla@vetmed.uni-giessen.de (C.H.); 4 Department of Infectious Disease Epidemiology, Bernhard-Nocht Institute for Tropical Medicine, 20359 Hamburg, Germany; julien.nguinkal@bnitm.de

**Keywords:** *Cryptosporidium*, diarrhea, genotype, children, Cameroon

## Abstract

Cryptosporidiosis remains a major cause of diarrhea-related childhood death, particularly in developing countries. Although effective anti-retroviral therapy has significantly reduced the cryptosporidiosis burden in western nations, the situation in developing countries remains alarming due to limited therapeutic options and a lack of preventive measures. To better control disease transmission and develop effective prevention strategies, a thorough understanding of the genetic diversity of circulating species is crucial. While cryptosporidiosis has previously been reported in Cameroon, information on the genetic diversity of parasite strains is lacking. In a large cross-sectional study conducted between March 2020 and March 2021 in four regions of Cameroon, Southwest, Littoral, Center and West, a total of 1119 fecal samples of children (*n* = 1119) were collected and genetically analyzed. This study aimed to assess the genetic diversity of *Cryptosporidium* strains circulating in this patient cohort in Cameroon. Using modified Ziehl–Neelsen fecal smear staining, an overall prevalence of 8.5% (96/1119) was recorded. PCR analysis revealed a prevalence of 15.4% in the Center, 7.2% in the Littoral, 10.5% in the West, and 13.1% in the Southwest regions. Molecular analysis identified *Cryptosporidium. hominis* and *Cryptosporidium parvum* as circulating species, with all subtype families suggesting anthroponotic transmission. No zoonotic subtypes of *C. parvum* were detected. These findings confirm that cryptosporidiosis transmission in Cameroon is primarily anthroponotic. Nonetheless, much larger epidemiological surveys, including other patient cohorts, are necessary for final confirmation of this statement.

## 1. Introduction

*Cryptosporidium* is an intestinal apicomplexan protozoan parasite of the Cryptosporiidae family within the phylum Alveolata and has been the cause of numerous water- and food-borne outbreaks of human and livestock enteric disease [1]. This parasite may produce a short-term and self-limiting illness in immunocompetent persons, clinically manifested by transient diarrhea, nausea, vomiting, fever, and abdominal pain [2,3]. Nonetheless, in immunocompromised patients, especially those with AIDS, or malnourished children, human cryptosporidiosis is characterized by a prolonged, life-threatening, and cholera-like diarrhea [4,5,6] Furthermore, infective *Cryptosporidium* oocysts are known to resist chlorination and thus are abundantly present in surface waters. *Cryptosporidium hominis* and *Cryptosporidium parvum* are the most important species infecting humans worldwide [3,7,8]. While anthroponotic *C. hominis* seems to mainly infect humans, *C. parvum* is zoonotic and infects a broad range of hosts, including humans and bovines. Especially in neonatal calves, it causes watery diarrhea resulting in significant economic losses for farmers and environmental oocyst contamination [9]. So far, preventive approaches, like vaccines, are completely lacking. Moreover, treatment of cryptosporidiosis in humans exclusively relies on nitazoxanide, which shows poor efficacy in immunocompromised patients and malnourished children, i.e., the main risk groups for human cryptosporidiosis [10].

The overall importance of human cryptosporidiosis in developing countries was highlighted by a global study covering all enteric diseases, ranking human cryptosporidiosis second to rotavirus infections concerning diarrhea in children [11]. While the use of highly active anti-retroviral therapies (HAARTs) against HIV infection has significantly reduced the burden of human cryptosporidiosis in industrialized countries, it is still a major cause of diarrhea in neonates and children, especially under two years of age, in developing countries [12,13,14]. Several subtypes of *Cryptosporidium* spp. have been associated with outbreaks of water-borne diseases in industrialized countries, like Korea [15], New Zealand [16], Hungary [17] and, more recently, the United Kingdom (Crypto Outbreak in Devon, UK, May 2024, BBC). In the USA, recreational water was associated with 156 of 444 (35.1%) reported cryptosporidiosis outbreaks from 2009 to 2017 [18].

*Cryptosporidium* species differ in host range and parasite–host coevolution, host adaptation, epizootiology, transmission, as well as geographic segregation, leading to the formation of subtype families with unique phenotypic traits within the major human–pathogenic species *C. parvum* and *C. hominis* [8,19,20]. In human populations, the most common species correspond to the anthroponotic species *C. hominis.* followed by the zoonotic species *C. parvum* [21]. However, only *C. parvum* can be cultured in vitro, driving it to become the focus of many experimental models used for basic research [22,23,24], vaccine and drug development [25]. Even though different *C. parvum* isolates from humans, farm animals, companion animals, and rodents are morphologically and developmentally similar, differences in host specificity, pre-patency, patency, and pathogenicity were documented [26,27,28,29]. Overall, more than 20 *Cryptosporidium* species and genotypes have been identified so far in humans [30] and a total of 44 *Cryptosporidium* species and over 120 wildlife genotypes described to date [31]. Identification of *Cryptosporidium* at the species or genotype level is essential for the assessment of infection sources and public health implications driven by parasite maintenance in domestic animals. Transmission intensity, genetic diversity, occurrence of genetic recombination and selective pressure have further shaped the parasite population’s genetic structures. Thus, the distribution of *Cryptosporidium* species in humans differs between developing and industrialized countries, with *C. hominis* dominating in developing countries, whilst both *C. hominis* and *C. parvum* are more or less equally present in industrialized nations [32,33,34]. These two species are responsible for almost all human cryptosporidiosis outbreaks reported so far [35]. However, there is a lack of information on the genetic diversity of *Cryptosporidium* amongst human populations, especially for developing countries.

*Cryptosporidium* spp. have been diagnosed in nearly all warm-blooded animals investigated so far [36,37,38,39] and consequently are present in both terrestrial and marine ecosystems [3,40,41,42,43,44]. The ubiquitous nature of this parasite makes it a serious threat to global health and emphasizes the urgent need to establish a comprehensive genotype mapping of *Cryptosporidium*, both geographically and across the different terrestrial and marine hosts. Importantly, given that efforts to develop a vaccine against cryptosporidiosis are ongoing, genetic diversity studies are also pivotal for optimized and efficient application of any protective vaccine.

Among the methods for genotypic analysis of *Cryptosporidium*, DNA sequencing of selected gene loci and restriction fragment length polymorphism analysis (RFLP) are the most popular. Reference genes, such as 18S rRNA, COWP, HSP70 and TRAP, have all successfully been applied to identify and classify *Cryptosporidium* spp. [45]. In addition, the highly polymorphic GP60 gene, which seems linked to pathogenicity [46,47], is preferentially used for sub-species genotyping.

In Cameroon, the burden of cryptosporidiosis in humans and livestock, as well as its transmission pattern, have been barely investigated. More importantly, Cameroonian *Cryptosporidium* spp. genetic diversity remains entirely unknown. Few studies have been conducted on the occurrence of cryptosporidiosis in HIV patients [48,49] and on environmental oocyst contamination of streams [50]. However, these studies all used microscopy for oocyst detection, which does not give any information on the genetic diversity of the species, thereby emphasizing the need for more in-depth studies in Cameroon. Referring to other regions of the African continent, a number of studies have been published so far, which include the molecular characterization of *Cryptosporidium* species, with examples from Ghana [51] Nigeria [52], Egypt [53], Gabon, Ghana, Madagascar and Tanzania [54], Zambia [55], Mozambique [56], Kenya [57], São Tomé and Principe [58], South Africa [59] and Uganda [60].

The aim of the current study was to determine the genetic diversity of *Cryptosporidium* spp. circulating among risk groups in Cameroon.

## 2. Materials and Methods

### 2.1. Sample Collection

**Figure 1:**
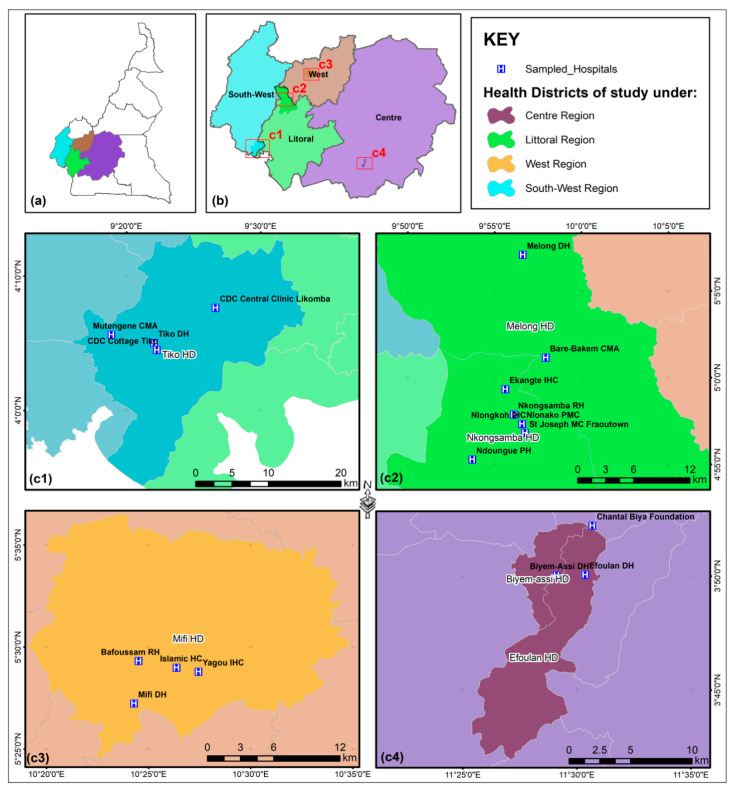
Geographical map illustrating stool sample collection sites in Cameroon. (**a**) Represents the map of Cameroon; the four regions sampled (**b**) are projected in more detail to show the individual health centers from which samples were collected (**c1**–**c4**).

In a cross-sectional study conducted between March 2020 and March 2021, fecal samples (*n* = 1119) were collected from children aged zero to ten years from four regions of Cameroon: Southwest, Littoral, Center and West (Figure 1). Participants included in the study were hospitalized patients (in- and outpatients) who presented with symptoms of loose, diarrheic stools (at least 3/day) and vomiting (at least once within the past 24 h). Patients who did not fulfil these criteria were excluded from the study. All health facilities included were in urban areas.

### 2.2. Modified Ziehl Neelsen (MZN) Staining

Freshly collected stool samples were homogenized, then applied as moderately thick fecal smears on standard microscope glass slides, using a sterile applicator stick. The smears were allowed to air dry for 20 min at room temperature (RT) before staining. The smears were stained using the Ziehl Neelsen Method (MZN) [61] with slight modifications. Briefly, the slides were fixed in absolute methanol for 3 min followed by staining with strong carbolfuchsin (Sigma-Aldrich, Darmstadt, Germany) for 20 min. The slides were thoroughly rinsed with tap water followed by a minimal decolorization by rapid agitation in 3% hydrochloric acid-alcohol for 10 s followed by a quick rinse with tap water. The smear was counterstained for 30 s in 1% methylene blue (Sigma-Aldrich, Darmstadt, Germany), rinsed with tap water, and allowed to air dry. The stained slides were examined under a phase contrast CHBS Olympus microscope (Olympus, Hamburg, Germany) using the 100× oil immersion objective.

### 2.3. DNA Extraction

DNA was extracted from approximately 200 mg of each fecal sample by the alkali freeze–boil method. For each sample, 800 μL of alkali washing solution (0.5 M NaOH and 0.05 M sodium citrate) was added and the samples were mixed for 10 min at RT by inversion using a rotary wheel (60 rpm). Thereafter, samples were centrifuged three times at 13,000× *g* for 5 min. The cell pellet was re-suspended in 0.5 mL Tris-HCl (pH 8.0) and re-centrifuged at 13,000× *g* for 5 min. This step was repeated once and the resulting pellet was re-suspended in Tris-EDTA (0.1 mL, 10 mM Tris HCl, pH 8.0, containing 1 mM EDTA). DNA was extracted by 4 cycles of freezing and thawing (−80 °C for 45 min and 100 °C for 12 min), respectively. The resulting extract was centrifuged at 13,000 *g* for 15 min and the supernatant containing total DNA was transferred to a sterile 1.5 mL Eppendorf tube [62]. The extracted DNA was quantified using a NanoDrop spectrophotometer (Thermo Fisher Scientific, Waltham, MA, USA) at 260 nm and stored at −20 °C until PCR amplification.

### 2.4. SSU-rRNA Nested PCR

For SSU-rRNA nested PCR, gene specific primers were used as follows: outer primers [(5′TTCTGAGCTAATACATGCG 3′) and (5′CCCATTTCCTTCGAAACAGGA 3′)] and inner primers [(5’GGAAGGGTTGTATTTATTAGATAAAG-3′) and (5′-AAGGAGTAAGGAACAACCTCCA-3′)] [45]. The amplification conditions were set as follows: initial denaturation of 95 °C for 3 min followed by 35 cycles of a three-step program (94 °C for 45 s, 55 °C for 45 s and 72 °C for 1 min), and a final extension step at 72 °C for 7 min. The same reaction conditions were used for primary and secondary PCR. The secondary amplicons were separated on 1.2% agarose gels and visualized using a UV-trans illuminator BIO-RAD Molecular Imager© Gel Doc™ XR+ imaging system (BIORAD, Feldkirchen, Germany). Positive (*Cryptosporidium parvum* oocysts) and negative (containing no template DNA) controls were included in each assay.

### 2.5. Restriction Fragment Length Polymorphism (RFLP) Analysis

For the RFLP analysis, 10 U of either SspI or AseI enzymes were used to digest 10 μL of DNA (amplicon from the 18S rRNA PCR) at 37 °C in a water bath for 2 h, as recommended by the supplier (New England Biolabs Inc., Ipswich, MA, USA). The samples were then separated by gel electrophoresis using 2% agarose and visualized after ethidium bromide staining (5 mg/mL, Sigma-Aldrich) using a BIO-RAD Molecular Imager© Gel Doc™ XR+ imaging system.

### 2.6. Cryptosporidium GP60 Gene Amplification

A gene fragment of the 60 kDa glycoprotein (GP60) was amplified by a two-step nested PCR, as previously described [27]. Briefly, 2 µL of extracted DNA was used at a final reaction volume of 20 µL. The primary PCR step was performed with the forward (Fp) and reverse (Rp) primers (5′-ATAGTCTCCGCTGTATTC-3′) and (5′-GGAAGGAACGATGTATCT-3′), respectively, using a GeneAmp^®^ PCR System 9700 thermocycler (Applied Biosystems). The resulting amplicons were diluted 1:50 and used as template for secondary amplification with the primers (5′TCCGCTGTATTCTCA GCC-3′) and 5′GCAGAGGAACCAGCATC-3′). Cycling conditions were set as follows: initial denaturation at 95 °C for 3 min followed by 35 cycles of a three-step program (94 °C for 45 s, 50 °C for 45 s and 72 °C for 1 min), and a final extension step at 72 °C for 7 min. The secondary amplicons were separated on 1.2% agarose gels and visualized using a UV-trans illuminator BIO-RAD Molecular Imager^©^ Gel Doc™ XR+ imaging system.

### 2.7. DNA Sequencing and Phylogenetic Analysis

Secondary GP60 PCR products were purified using the quick DNA purification kit from Qiagen (QIAquick^®^ Gel Extraction kit, Qiagen, Hilden, Germany). Amplicons were subsequently sent for sequencing (Inqaba Biotec, Pretoria, South Africa). Direct DNA sequencing of GP60 amplicons purified from the gels was performed using the primers GP60-AL3532 (5′-TCCGCTGTATTCTCAGCC-3′) and GP60-AL3534 (5′-GCAGAGGAACCAGCATC-3′) on an ABI PRISM—3730 XL DNA Analyzer using the BigDye Terminator v3.1 Cycle Sequencing Kit (Applied Biosystems, Foster City, CA, USA). Sequence accuracy was confirmed by two-directional sequencing. The raw sequences obtained were edited with DNAstar Lasergene Editseq version 7.1.0 (http://www.dnastar.com/).

Raw sequence reads were initially assessed for quality using FastQC, then trimmed with fastp [63] to remove low-quality sequences. The quality threshold was set at Q20 to ensure 99% accuracy, and sequences shorter than 50 base pairs were discarded. High-quality sequences (*n* = 38) were aligned using MAFFT [64], and the resulting alignment was trimmed using trimAl [65] with the “automated1” option for low-quality alignment regions. A maximum likelihood phylogenetic tree was constructed using IQ-TREE [66], incorporating reference *GP60* sequences (*AY382670-Id*, *AY873783-Ia*, *GU214369-IIe*, *AY166807-Ib*, *FJ839876-IIc*, and *GU214365-IIc*) of *Cryptosporidium* spp. from NCBI. The best-fit model of nucleotide substitution was selected using ModelFinder Plus within IQ-TREE, and branch support was assessed with 1000 ultrafast bootstrap approximations.

### 2.8. Statistical Analysis

Statistical analyses were performed using R statistical package version 4.4.1 (www.r-project.org). Descriptive statistics were performed to characterize the study population. To identify determinants of *Cryptosporidium* infection in study regions, multiple logistic regression was fitted with PCR results (1 for positive and 0 for negative) as outcome variable, and children’s age, gender, duration of diarrhea episode, and the mothers’ education level as predictor variables. The regression analysis results were presented as adjusted odds ratios, associated confidence intervals, and *p*-values. The confidence level in this study was set at 95%.

## 3. Results

### 3.1. Demographic Data

Overall, a total of 1119 stool samples were collected from children in the geographic regions of the Center (*n* = 272), Southwest (*n* = 320), Littoral (*n* = 223) and West (*n* = 304) in Cameroon (Figure 1). Samples were assessed for the presence of *Cryptosporidium* by both microscopic analysis of MZN-stained fecal smears and PCR. For socio-demographic characteristics of the sample population, see Table 1.

### 3.2. Results of Microscopic Analysis

Microscopic examination revealed a total of 96 samples (96/1119) as positive for *Cryptosporidium* oocysts, thereby representing a total prevalence of 8.5%.

### 3.3. Results of PCR Analysis

**Figure 2:**
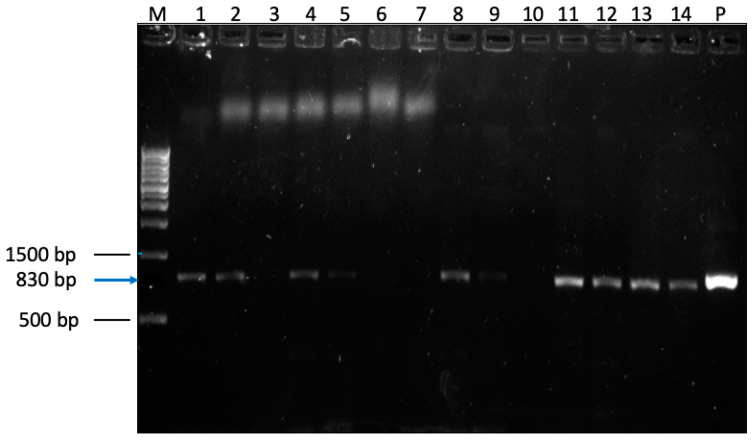
Secondary amplicons from *Cryptosporidium*-nested PCR of the 18S rRNA gene indicates amplicon sizes at 830 bp and no amplification in the negative control. M = DNA ladder, 1–14 = samples, P = positive control. Gene PCR gel map original image can be found in Figure S1.

Positive samples showed a typical amplicon size of 830 bp in agarose gels after nested PCR (Figure 2). Of the 1119 samples collected, 132 were positive for *Cryptosporidium*, giving an overall prevalence of 11.8%. Referring to different regions of Cameroon, the prevalence varied between 7.2% and 15.4%: Center region 15.4% (42/272); Littoral region 7.2% (16/223); West region 10.5% (32/304) and Southwest region 13.1% (42/320) (Table 2).

Different parameters, like age, gender, duration of diarrhea and educational level of the mothers, were also analyzed but showed no statistically significant differences (Table 2).

### 3.4. Cryptosporidium spp. Genotyping

**Figure 3:**
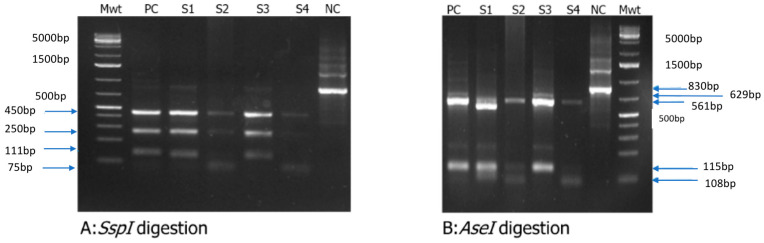
Restriction fragment length polymorphism (RFLP) analysis of *Cryptosporidium* 18S rRNA gene amplicons. (**A**) Ssp I digestion results in three bands of 450 bp, 250 bp and 111 bp size, which are typical for *Cryptosporidium* spp.; (**B**) Ase I digestion of the 18S rRNA gene amplicons resulted in typical bands sizes of 629 bp and 115 bp specific for *C. parvum* (S1 and S3) as well as of 561 bp and 108 bp specific for *C.hominis* (S2 and S4), Mwt = DNA ladder, PC = positive control, S1-S3 = samples 1 to 3, NC = negative control representing the undigested amplicon. Gene PCR gel map original image can be found in Figure S2.

Enzymatic digestion of 18S rRNA amplicons with SspI yielded multiple DNA fragments with band sizes of 75 bp, 111 bp, 250 bp and 450 bp (Figure 3A), which are in line with reports for *C. parvum* or *C. hominis* [67]. Likewise, digestion with AseI generated two DNA fragments of 561 bp and 629 bp specific for *C. hominis* and *C. parvum*, respectively (Figure 3B).

### 3.5. Cryptosporidium spp. Sub-Genotyping and Evolutionary Relationships of Taxa

**Figure 4:**
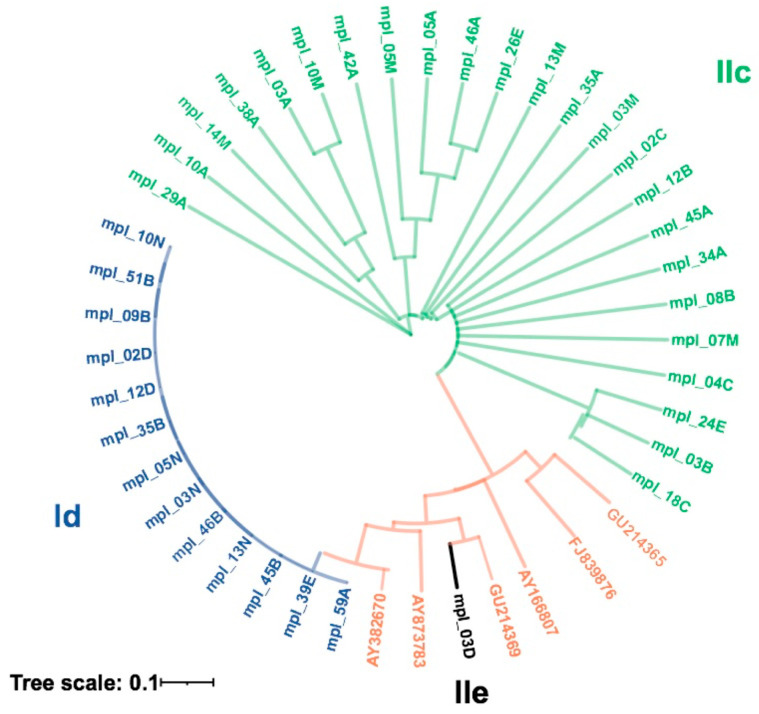
Phylogenetic analysis of *C. hominis* and *C. parvum* subtypes and reference strains (in red) using maximum likelihood of the glycoprotein 60 (*gp60*) gene. Reference sequences for the subtypes are AY382670-Id, AY873783-Ia, GU214369-IIe, AY166807-Ib, FJ839876-IIc, GU2143365-IIc.

The next step in the characterization of field isolates consisted in analyzing the sequence of the GP60 gene. Therefore, 38 isolates were selected from all positive samples, sequenced after amplification of the GP60 gene locus and compared with reference GP60 DNA sequences obtained from the NCBI database. From the 38 isolates, 13 belonged to the *C. hominis* subtype family Id, 24 to the *C. parvum* subtype family IIc, and one isolate to the subtype family IIe (Figure 4).

## 4. Discussion

Cosmopolitan outbreaks of human cryptosporidiosis have regularly been linked to zoonotic transmission resulting from human contact with infected domestic and wildlife animals [68]. Despite this epizootiological fact, very few studies dealing with *Cryptosporidium* species/genotypes and their origin exist for Africa, where human cryptosporidiosis is a major cause of pediatric morbidity and mortality [67,68,69]. Thus, detailed knowledge of genotypes/sub-genotypes of *Cryptosporidium* species circulating in a defined human population will help to understand transmission routes in order to clarify if oocyst sources originate from zoonotic or anthroponotic origin. Additionally, knowledge of *Cryptosporidium* genotypes seems relevant for the design of optimal control programs by national public health authorities for human protection or even eradication of human cryptosporidiosis, as postulated elsewhere [67,69].

Multiple studies in sub-Saharan Africa have revealed a high prevalence of cryptosporidiosis in children and immunocompromised patients dominated by two species, *C. hominis* and *C. parvum*. *C. parvum* bears a high veterinary importance, particularly for neonatal livestock (e. g. calves, lambs, goat kids), and is considered as one of the most relevant etiological agent of neonatal enteritis [70,71,72]. Moreover, *C. parvum* occurs in a wide spectrum of terrestrial mammalian wildlife, including wild horses, deer, mountain gorillas, nutrias, raccoon dogs and camelids, among others [70,73]. Wildlife represents, therefore, an important natural reservoir and adds—together with humans and livestock—to the pool of *C. parvum* genotypes within the environment [69]. However, very few studies have characterized the genotypes or subtypes of human cryptosporidiosis in African countries [12,68,69].

With the current epidemiological study, we have shown a *Cryptosporidium* prevalence of 11.8% in children from 0 to 10 years of age for Cameroon by PCR analysis, representing a total of 132 positive children (*n* = 132/1119) from four different geographic regions of this West-African country. Although these results cannot be extrapolated to the nationwide situation, it is a representative distribution, since the four regions sampled represent a substantial fraction of the total population of the country. As expected, the current results further confirmed that PCR methodology is slightly more sensitive than MZN-fecal staining (11.8 % vs. 8.5 %), even though the difference is not statistically significant. Others have reported similar results, also with no statistically significant difference [21,74,75]. When considering the age-related sample distribution, the samples were divided in two groups, i.e., children below 5 years and children from 5 to 10 years. We observed a slightly higher prevalence of *Cryptosporidium* in children below 5 years (12%) compared to children aged 5 to 10 (7%), although this difference was not statistically significant. Furthermore, the consideration of symptoms, such as the duration of diarrhea, revealed that children with diarrhea for more than 2 days were slightly more likely to be *Cryptosporidium*-infected (12%) than those who had diarrhea for 2 days or less (8%). However, this difference was again not statistically significant. A meta-analysis of cryptosporidiosis in children under 5 years estimated this parasitic infection to be related, with 44.8 million diarrheal episodes and 48,300 deaths, globally [76]. In the present study, it seems likely that lack of a clear difference between the symptomatic (diarrhea for more than 2 days) and asymptomatic (diarrhea for 2 days or less) group is due to the inclusion of children up to 10 years, who might already have developed a parasite-specific T cell response. Hence, immunocompetent *C. parvum* re-infected children are more likely to develop a CD8^+^ T cell-based protective immunity to homologous infections, resulting in marginal intestinal damage and/or subclinical cryptosporidiosis [77,78,79].

The phylogenetic analysis of a representative number of isolates revealed that a substantial number belong to the subtype families Ia, Ib and Id, representing the anthroponotic *C. hominis*. Interestingly, these subtype families have been associated with human-to-human transmission in children and HIV-positive adults in several developing countries [35,80,81]. The largest group of isolates from this study belonged to the subtype family IIc, which represents the anthroponotic *C. parvum*. One sequence belonged to the subtype family IIe, which is also an anthroponotic *C. parvum*. Hence, these two anthroponotic subtype families have only been found in human specimens so far [51,82]. Strikingly, the zoonotically transmitted subtype families of *C. parvum*, i.e., IIa, IId and IIl, were not found in any of the current isolates. This study elucidates for the first time the genetic diversity of *Cryptosporidium* species circulating among Cameroonian children and supports the previously reported tendency that transmission of cryptosporidiosis in Africa is essentially based on human-to-human transfer, since all subtype families from both *C. hominis* and *C. parvum* identified in this study are anthroponotic subtypes. 

Some limitations of this study were the fragmentary coverage of the entire country and the fact that not all isolates were sequenced, but were rather a representative fraction. Moreover, future identification analysis of the species may also include 18S rRNA gene sequencing to improve the robustness of the result. A larger study population covering a wider geographical area of Cameroon would certainly improve the robustness of these results.

## 5. Conclusions

From this study, it appears that cryptosporidiosis transmission in Cameroonian children is likely to proceed predominantly through the anthroponotic route, since no zoonotic subtypes were found. It is therefore plausible that implementation of a better control strategy to prevent human-to-human transmission would lead to a significant impact in the reduction of the overall disease burden.

## Figures and Tables

**Table 1:** Socio-demographic characteristics of the population.

	Regions *n* (%)				
Variables	Center	Southwest	Littoral	West	Overall *n* (%)
**Age**					
<5 yrs	248 (91.18)	150 (46.88)	179 (80.27)	189 (62.17)	766 (68.45)
5–10 yrs	24 (8.82)	170 (53.12)	44 (19.73)	115 (37.83)	353 (31.55)
**Gender**					
Female	119 (43.75)	174 (54.37)	95 (42.60)	136 (44.74)	524 (46.83)
Male	153 (56.25)	146 (45.62)	128 (57.40)	168 (55.26)	595 (53.17)
**Duration of Diarrhea (days)**					
<2	61 (22.43)	98 (30.63)	97 (43.50)	107 (35.20)	363 (32.44)
>2	211 (77.57)	222 (69.38)	126 (56.50)	197 (64.80)	756 (67.56)
**Education of mother**					
Primary	30 (11.03)	100 (31.25)	46 (20.63)	52 (17.11)	228 (20.38)
Secondary	242 (88.97)	209 (65.31)	139 (62.33)	241 (79.28)	831 (74.26)
Tertiary	0	11 (3.44)	38 (17.04)	11 (3.62)	60 (5.36)

**Table 2:** Prevalence of cryptosporidiosis based on PCR.

Variables	Number Positive	Total Examined	Prevalence (%)	
**Age**				
<5 yrs	94	766	12.27	
5–10 yrs	38	353	10.76	
**Gender**				
Female	66	524	12.60	
Male	66	595	11.09	
**Duration of Diarrhea (days)**				
<2	38	363	10.47	
>2	94	756	12.43	
**Education of Mother**				
Primary	24	228	10.53	
Secondary	102	831	12.27	
Tertiary	6	60	10.00	
**Overall Prevalence**				
Number of positive	Total Examined	Prevalence	Lcl	Ucl
132	1119	11.8	10.03	13.82
	(Result = “Positive”)			
Predictors	Odds Ratios	CI	*p*	
(Intercept)	0.23	0.12–0.43	<0.001	
Age [5–10 yrs]	0.83	0.55–1.24	0.365	
Gender [Male]	0.83	0.57–1.20	0.317	
Duration of Diarrhea [>2]	1.23	0.83–1.86	0.321	
Education [Secondary]	1.28	0.81–2.11	0.308	
Education [Tertiary]	0.98	0.35–2.41	0.968	
Observations	1119			

## Data Availability

All data generated or analyzed during the study are included in this published article. All sequences will be submitted to CrytoDB upon publication.

## Supplementary Materials

The following supporting information can be downloaded at: https://www.mdpi.com/article/10.3390/pathogens14030287/s1, Figure S1: Gene PCR gel map original image of Figure 2; Figure S2: Gene PCR gel map original image of Figure 3.
