# Supplementary material for: Prevalence and Molecular Characterization of Cryptosporidium Species in Diarrheic Children in Cameroon
Source: Pathogens. 2025 Mar 14;14(3):287. doi: 10.3390/pathogens14030287 (PMC11945035; doi:10.3390/pathogens14030287)
Supplement: Supplementary file 1 [file pathogens-14-00287-s001.zip › pathogens-3485051-supplementary.pdf]

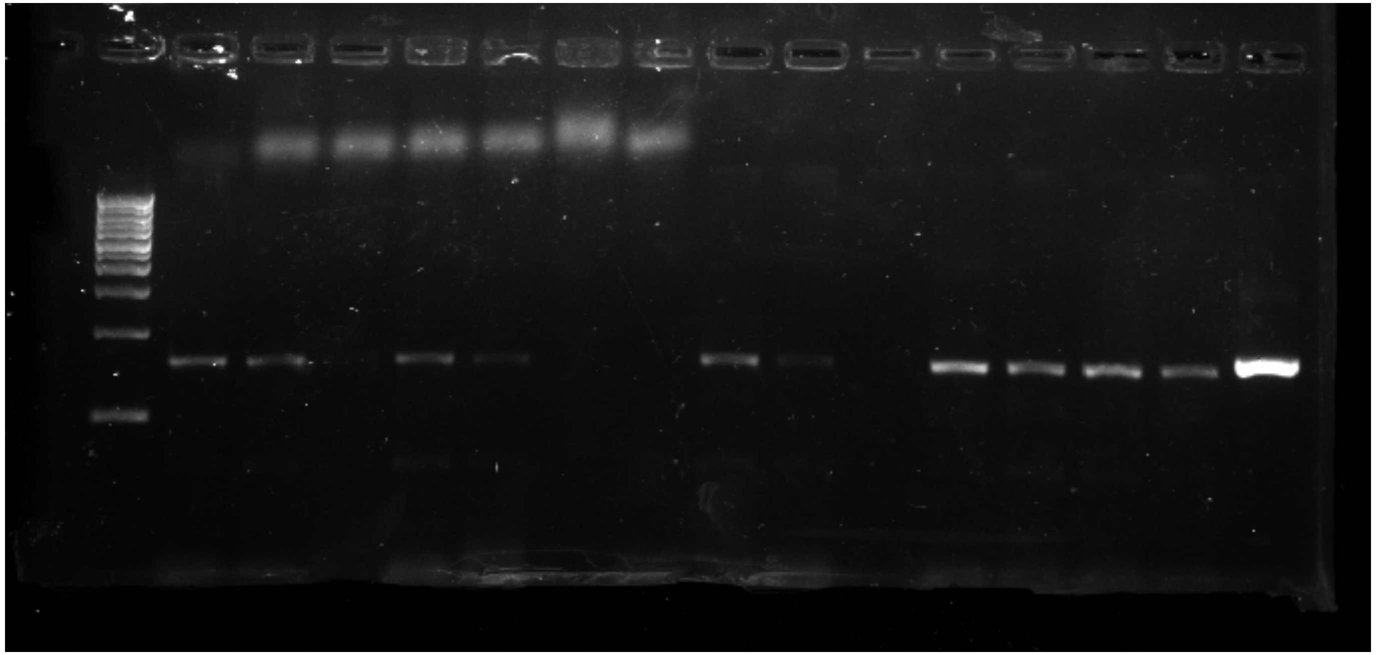

Figure S1. Gene PCR gel map original image of Figure 2.

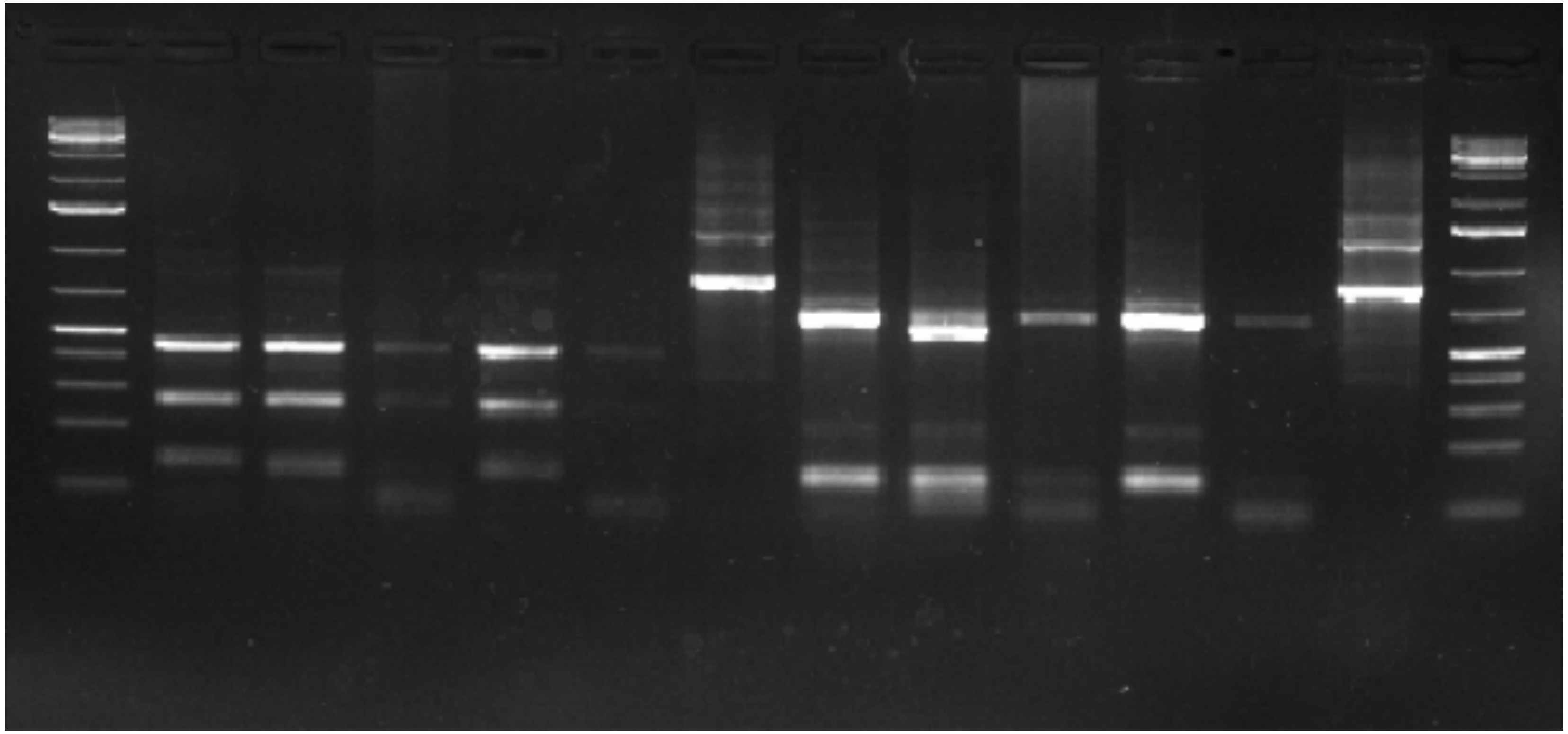

Figure S2. Gene PCR gel map original image of Figure 3.
